# Gender-related differences in cannabis use in schizophrenia patients before and after antipsychotic treatment

**DOI:** 10.1192/j.eurpsy.2025.2260

**Published:** 2025-08-26

**Authors:** A. Rojs, U. M. Battelino, U. Ogrizek, A. Čelofiga

**Affiliations:** 1 Maribor University Medical Centre; 2 Faculty of Medicine University of Maribor; 3 Department of Psychiatry, Maribor University Medical Centre, Maribor, Slovenia

## Abstract

**Introduction:**

Cannabis is the most widely used psychoactive substance among youth, and its use has been increasingly linked to psychiatric disorders, particularly psychosis. THC, the psychoactive component of cannabis, has the potential to trigger or worsen schizophrenia. Cannabis use is associated with an earlier onset of schizophrenia and is more prevalent among males with first-episode psychosis, which may contribute to an earlier onset of schizophrenia observed in men. With increasing cannabis legalization, understanding gender differences in cannabis use in schizophrenia patients is essential for personalized treatment approaches.

**Objectives:**

To examine the gender-specific impact of cannabis use on schizophrenia or other psychotic disorders, focusing on pre-onset use and discontinuation following antipsychotic (AP) treatment.

**Methods:**

A retrospective study was conducted including personal interviews and medical history reviews of inpatients and outpatients. Inclusion criteria were age between 18 and 65 years, diagnosis of schizophrenia or schizoaffective disorder, and use of AP therapy, both for at least 5 years. Statistical analysis was performed using the Jamovi statistical analysis software.

**Results:**

136 patients being treated at University Medical Centre Maribor’s Department of Psychiatry were included in the study. 38.2% of patients were females and 61.1% were males. The mean age of all participants was 49.2±11.8 years. The mean age at the time of diagnosis was statistically significantly higher in females compared to males (30.4±8.9 versus 26.0±7.9 years; Mann Whitney U test: p<0.001). Before the onset of psychosis, 23.1% females and 56.0% males used cannabis, while 76.9% females and 44.0% males did not (Image 1). A chi-squared test showed a significant gender difference in cannabis use (p<0.001), indicating higher prevalence among males. After introduction of the AP treatment 58.3% females and 57.4% males discontinued cannabis use (Image 2). A chi-squared test indicated no significant association between sex and cannabis discontinuation (p=0.956), with similar rates for both genders.

**Image 1:**

**Figure:**
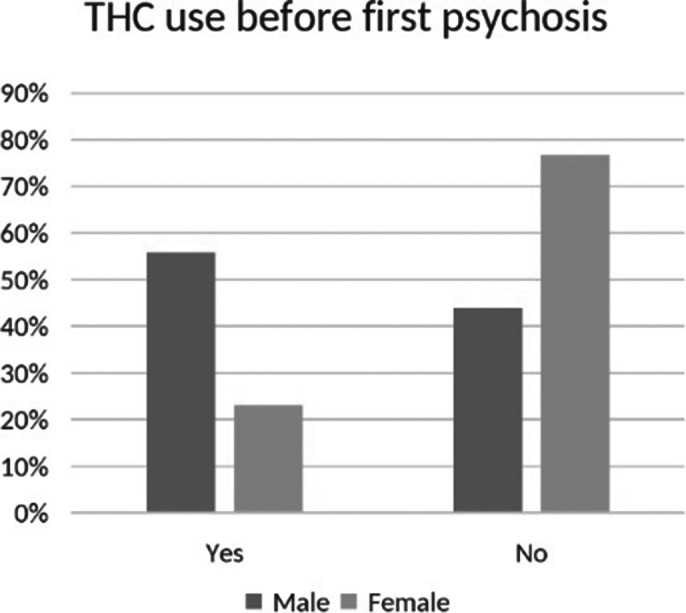

**Image 2:**

**Figure:**
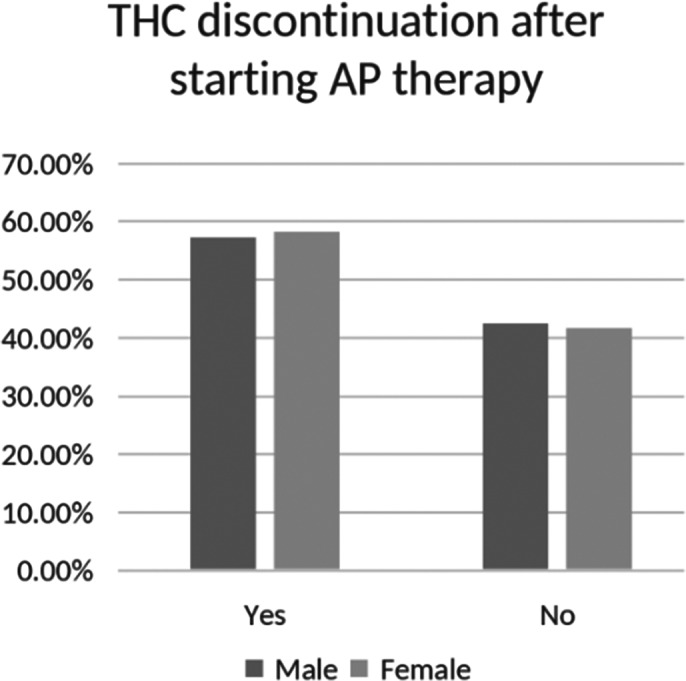

**Conclusions:**

Our study reveals significant gender differences in cannabis use before psychosis onset, with males showing higher prevalence and potentially earlier diagnosis. However, cannabis discontinuation rates after AP treatment were similar across genders, suggesting comparable responses to treatment. As cannabis legalization increases, the need for customized interventions that consider gender-specific factors in managing psychotic disorders is emphasized. Further research is necessary to investigate the long-term effects of cannabis use on treatment outcomes in both males and females.

**Disclosure of Interest:**

None Declared

